# Optimizing periprosthetic fracture management and in-hospital outcome: insights from the PIPPAS multicentric study of 1387 cases in Spain

**DOI:** 10.1186/s10195-024-00746-6

**Published:** 2024-03-07

**Authors:** The PIPPAS Study Group, Héctor J. Aguado, Pablo Castillón-Bernal, Jordi Teixidor-Serra, Yaiza García-Sánchez, Josep M. Muñoz-Vives, Pilar Camacho-Carrasco, Montsant Jornet-Gibert, Cristina Ojeda-Thies, Pablo García-Portabella, Adela Pereda-Manso, Elvira Mateos-Álvarez, Javier Manzano-Mozo, Raquel Carrillo-Gómez, Sergio País-Ortega, Virginia García-Virto, David Noriega-González, Begoña Aránzazu Álvarez-Ramos, Abel Ganso-Pérez, Carmen Cervera-Díaz, María Plata-García, Alina Ortega-Briones, Juan Berrocal-Cuadrado, Diego Criado del Rey-Machimbarrena, Jordi Salvador, Laura Rey, Jordi Tomás-Hernández, Jordi Selga-Marsà, José Vicente Andrés-Peiró, Jordi Querolt-Coll, Guillermo Triana, Marian Vives-Barquiel, Marina Renau-Cerrillo, Borja Campuzano-Bitterling, José M Hernández, Ricardo Ostilla, Anna Carreras-Castañer, Pere Torner, Rebeca Díaz-Suárez, Eliam Ajuria Fernández, Carlos Olaya-González, María Fernández-Villán, Unai García de Cortázar, Mirentxu Arrieta, Daniel Escobar, Estíbaliz Castrillo, Patricia Balvis, Mónica Rodríguez-Arenas, Ángela García-Pérez, Jesús Moreta, Iñigo Bidea, Xabier Jiménez-Urrutia, Beatriz Olías-López, Juan Boluda-Mengod, David González-Martín, Leopoldo Bárcena-Goitiandia, Daniel López-Dorado, Juan Carlos Borrás-Cebrián, David García-Aguilera, Patricio Andrés Freile-Pazmiño, Miguel Ángel Suárez-Suárez, Lucía Lanuza-Lagunilla, Antonio García-Arias, Jaime Sánchez-Saz, Javier García-Coiradas, José Valle-Cruz, Jesús Mora-Fernández, María Ángeles Cano-Leira, Guillermo Rieiro, Antonio Benjumea-Carrasco, Rodrigo Jesús Priego-Sánchez, Coral Sánchez-Pérez, Jorge Guadilla-Arsuaga, Alexis Fernández-Juan, Plácido Sánchez, Javier Ricón, Alfonso Fuentes-Díaz, Elena M. García-García, Francisco Cuadrado-Abajo, Gonzalo García-Portal, Pedro del PozoManrique, Virginia Castillo del Pozo, Francisco Manuel Garcia-Navas, Ester García-Paredero, Teresa Beteta-Robles, Ainhoa Guijarro-Valtueña, Gonzalo Gutiérrez-Baiget, Noelia Alonso-García, Inés Navas-Pernía, Diana Ariza-Herrera, Joan Vilanova, Miquel Videla-Cés, Teresa Serra-Porta, César Vázquez-García, Carmen Carrasco-Becerra, Silvia Pena-Paz, Víctor Otero-Naveiro, Inés Fernández-Billón-Castrillo, Amaia Martínez-Menduiña, Carolina Hernández-Galera, Fátima Fernández-Dorado, María Madrigal-López, Antonio Murcia-Asensio, Elena Galián-Muñoz, Ángel Castro-Sauras, Teresa Espallargas-Doñate, María Royo-Agustín, Nuria Plaza-Salazar, Carla Gámez-Asunción, Adrián Muñoz-Vicente, Teresa Pareja-Sierra, Jennifer Benito-Santamaría, Alejandro Cuenca-Copete, Ana Verdejo-González, Blas González-Montero, Luis Alejandro Giraldo-Vegas, Laura Alonso-Viana, Eduardo José Díez-Pérez, Ricardo Briso-Montiano, Ana Isabel Andrés, Juan Mingo-Robinet, María Naharro-Tobío, Emma Escudero-Martínez, Jorge Serrano-Sanz, J. M. Peñalver-Matamoros, Núria Fernàndez-Poch, Laia Martínez-Carreres, María Macho-Mier, Carlos Martín-Hernández, Antonio Francisco Laclériga-Giménez, José Carlos Saló-Cuenca, César Salamanca-Ontiveros, Jordi Espona-Roselló, Victoria Altemir-Martínez, Guillermo Criado-Albillos, Jorge Cunchillos-Pascual, Mercedes Millán-Cid, Hugo Gabriel Cabello-Benavides, Jorge Martínez-Íñiguez-Blasco, Paloma Sevilla-Ortega, Juan Ramón Cano, Alicia Ramírez, Fernando Marqués-López, Santos Martínez-Díaz, Guido S. Carabelli, Pablo A Slullitel, Ignacio Astore, Bruno R. Boietti, Carlos Hernández-Pascual, Javier Marín-Sánchez, Julio César Córdova-Peralta, Iván Dot-Pascuet, Eduardo Pereira-Mosquera, Javier Martín-Antúnez, José María Pérez, Alfonso Mandía-Martínez, Julio De Caso, Jordi Martín-Marcuello, Miguel Benito-Mateo, A. David Murillo-Vizuete, Luis Gracia Delgado, Gaspar dela Herrán, Nahikari Nunes, Ivan Pérez-Coto, María Rosa González-Panisello, Susana Iglesias-Fernández, Gorka Luis Ruete-Gil, Sergio Ramos-García, Juan Pablo Villarreal

**Affiliations:** 1 Servicio de Traumatología, Hospital Clínico Universitario de Valladolid, Avenida de Ramón y Cajal 3, 47003 Valladolid, Spain; 2 Trauma department, Hospital Universitari Mútua de Terrassa, Barcelona, Spain; 3 Trauma Department, Hospital Universitari Vall d ´Hebrón, Barcelona, Spain; 4 Trauma Department, Hospital Fundació Althaia de Manresa, Barcelona, Spain; 5 Trauma department, Hospital Clínic de Barcelona, Barcelona, Spain; 6 Trauma Department, Hospital 12 de Octubre, Madrid, Spain; 7 Trauma department, Hospital de Jove, Gijón, Spain; 8 Trauma department, Hospital Universitario de Basurto, Bizkaia, Spain; 9 Trauma department, Hospital Álvaro Cunqueiro de Vigo, Pontevedra, Spain; 10 Trauma department, Hospital Universitario de Galdakao-Usansolo, Bizkaia, Spain; 11 Trauma Department, Hospital Universitario de Canarias, Tenerife, Spain; 12 Trauma Department, Hospital Infanta Elena de Valdemoro, Madrid, Spain; 13 Trauma department, Hospital Universitario Dr. Peset, Valencia, Spain; 14 Trauma department, Hospital Royo Villanova, Zaragoza, Spain; 15 Trauma department, Hospital Universitario de Cabueñes, Gijón, Spain; 16 Trauma Department, Hospital Clínico San Carlos, Madrid, Spain; 17 Trauma department, Complejo Hospitalario Universitario de A Coruña, A Coruña, Spain; 18 Trauma department, Hospital General Universitario Gregorio Marañón , Madrid, Spain; 19 Trauma department, Hospital Universitario de Álava, Vitoria, Spain; 20 Trauma department, Hospital General Universitario Los Arcos del Mar Menor, Murcia, Spain; 21 Trauma Department, Hospital Vega Baja de Orihuela, Alicante, Spain; 22 Trauma department, Hospital General Universitario J.M. Morales Meseguer, Murcia, Spain; 23 Trauma department, Hospital Universitario Marqués de Valdecilla, Santander, Spain; 24 Trauma department, Hospital Universitario de Toledo, Toledo, Spain; 25 Trauma department, Hospital Puerta de Hierro de Majadahonda, Madrid, Spain; 26 Trauma department, Complejo Asistencial de Segovia, Segovia, Spain; 27 Trauma department, Consorci Sanitari Integral - Hospital Sant Joan Despí- Moisès Broggi , Barcelona, Spain; 28 Trauma department, Hospital Universitari Sagrat Cor - Quirónsalud, Barcelona, Spain; 29 Trauma department, Complejo Hospitalario de Llerena-Zafra, Badajoz, Spain; 30 Trauma department, Hospital Universitario Lucus Augusti, Lugo, Spain; 31 Trauma department, Hospital Ramón y Cajal, Madrid, Spain; 32 Trauma department, Hospital Príncipe de Asturias, Alcalá de Henares, Spain; 33 Trauma department, Hospital General Universitario Reina Sofía, Murcia, Spain; 34 Trauma department, Hospital General Obispo Polanco, Teruel, Spain; 35 Trauma department, Hospital Universitario de Guadalajara, Guadalajara, Spain; 36 Trauma department, Hospital Universitari Doctor Josep Trueta, Girona, Spain; 37 Trauma department, Complejo Hospitalario Universitario de Albacete, Albacete, Spain; 38 Trauma department, Hospital Sierrallana de Torrelavega, Cantabria, Spain; 39 Trauma department, Complejo Asistencial Universitario de Palencia, Palencia, Spain; 40 Trauma department, Complexo Hospitalario Universitario de Pontevedra, Pontevedra, Spain; 41 Trauma department, Hospital Universitari Parc Taulí de Sabadell, Barcelona, Spain; 42 Trauma department, Hospital Universitario Miguel Servet, Zaragoza, Spain; 43 trauma Department, Hospital Universitario Arnau de Vilanova, Lleida, Spain; 44 Trauma department, Complejo Asistencial Universitario de Burgos, Burgos, Spain; 45 Trauma department, Hospital Universitario San Pedro, Logroño, Spain; 46 Trauma department, Hospital Universitario Costa del Sol de Marbella, Málaga, Spain; 47 Trauma department, Hospital Italiano, Buenos Aires, Argentina; 48 Trauma department, Hospital Parc De Salut Mar, Barcelona, Spain; 49 Trauma department, Hospital Universitario de Salamanca, Salamanca, Spain; 50 Trauma department, Hospital Universitari Sant Joan de Reus, Tarragona, Spain; 51 Trauma department, Hospital Virgen del Rocío, Sevilla, Spain; 52 Trauma department, Hospital de la Santa Creu i Sant Pau, Barcelona, Spain; 53 Trauma department, Hospital Universitario Infanta Leonor, Madrid, Spain; 54 Trauma department, Hospital Alto Guadalquivir de Andújar, Jaén, Spain; 55 Trauma department, Hospital Universitario de Donostia, San Sebastian, Spain; 56 Trauma department, Hospital Carmen y Severo Ochoa de Cangas del Narcea, Asturias, Spain; 57 Trauma department, Hospital Reina Sofía de Tudela, Navarra, Spain; 58 Trauma department, Hospital San Agustín de Avilés, Asturias, Spain; 59 Trauma department, Hospital Universitario Virgen Macarena, Sevilla, Spain

**Keywords:** Periprosthetic fracture, Outcome, Mortality, Replacement, Fracture fixation, Geriatric co-management, Incidence, Epidemiology, Management, Frailty

## Abstract

**Background:**

The incidence of all periprosthetic fractures (PPF), which require complex surgical treatment associated with high morbidity and mortality, is predicted to increase. The evolving surgical management has created a knowledge gap regarding its impact on immediate outcomes. This study aimed to describe current management strategies for PPF and their repercussions for in-hospital outcomes as well as to evaluate their implications for the community.

**Methods:**

PIPPAS (Peri-Implant PeriProsthetic Survival Analysis) was a prospective multicentre observational study of 1387 PPF performed during 2021. Descriptive statistics summarized the epidemiology, fracture characteristics, management, and immediate outcomes. A mixed-effects logistic regression model was employed to evaluate potential predictors of in-hospital mortality, complications, discharge status, and weight-bearing restrictions.

**Results:**

The study encompassed 32 (2.3%) shoulder, 4 (0.3%) elbow, 751 (54.1%) hip, 590 (42.5%) knee, and 10 (0.7%) ankle PPF. Patients were older (median 84 years, IQR 77–89), frail [median clinical frailty scale (CFS) 5, IQR 3–6], presented at least one comorbidity [median Charlson comorbidity index (CCI) 5, IQR 4–7], were community dwelling (81.8%), and had outdoor ambulation ability (65.6%). Femoral knee PPF were most frequently associated with uncemented femoral components, while femoral hip PPF occurred equally in cemented and uncemented stems. Patients were managed surgically (82%), with co-management (73.9%), through open approaches (85.9%) after almost 4 days (IQR, 51.9–153.6 h), with prosthesis revision performed in 33.8% of femoral hip PPF and 6.5% of femoral knee PPF. For half of the patients, the discharge instructions mandated weight-bearing restrictions. In-hospital mortality rates were 5.2% for all PPF and 6.2% for femoral hip PPF. Frailty, age > 84 years, mild cognitive impairment, CFS > 3, CCI > 3, and non-geriatric involvement were candidate predictors for in-hospital mortality, medical complications, and discharge to a nursing care facility. Management involving revision arthroplasty by experienced surgeons favoured full weight-bearing, while an open surgical approach favoured weight-bearing restrictions.

**Conclusions:**

Current arthroplasty fixation check and revision rates deviate from established guidelines, yet full weight-bearing is favoured. A surgical delay of over 100 h and a lack of geriatric co-management were related to in-hospital mortality and medical complications. This study recommends judicious hypoaggressive approaches. Addressing complications and individualizing the surgical strategy can lead to enhanced functional outcomes, alleviating the economic and social burdens upon hospital discharge.

*Level of Evidence* Level IV case series.

*Trial registration*: registered at ClinicalTrials.gov (NCT04663893), protocol ID: PI 20-2041.

**Graphical abstract:**

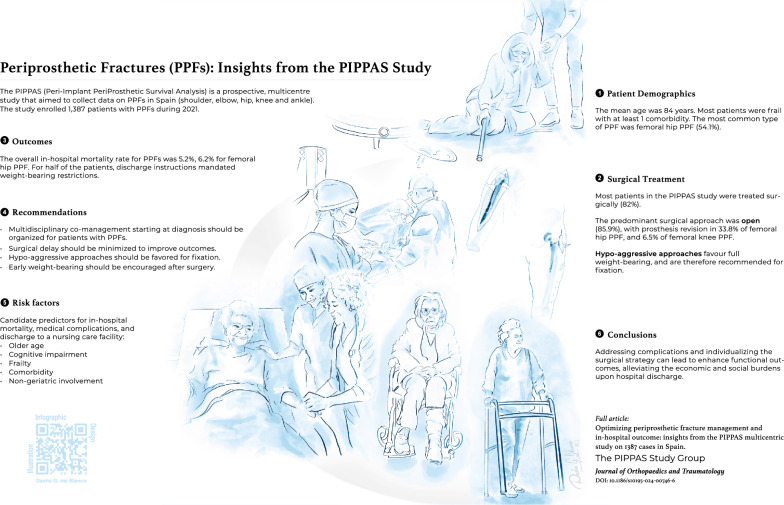

**Supplementary Information:**

The online version contains supplementary material available at 10.1186/s10195-024-00746-6.

## Introduction

Surgical treatment of periprosthetic fractures (PPF) presents challenges due to factors such as the prosthesis, bone defects, remodelling, and osteoporosis, which may hinder reduction and fixation [1–3]. Operative strategies aim to maintain joint/prosthesis functionality and typically involve either revising the joint with a new prosthesis or utilizing specifically adapted fixation devices [4]. When treating PPF, an assessment of the stability of the prosthesis is recommended; if it is loose, revision should be considered [5]. For a well-fixed prosthesis, fixation is preferred. However, an ongoing debate persists regarding whether to revise a loose prosthesis or retain it and fix the fracture, especially in very frail patients [2, 6–9]. Surgical technique and perioperative care significatively impact mortality and outcomes in PPF cases [1, 10]. Most of the studies available on this topic are retrospective, based on small single-hospital cohorts, cover extended time periods, and primarily focus on femoral PPF [1, 11], with limited data available on other locations.

Published studies encompass a wide range of surgical options for treating these fractures, which have evolved substantially in recent years. Retrospective research with outdated data may lack relevance and pertinence. Systematic reviews and meta-analysis highlight the need for prospective research, including registries and trials evaluating the outcomes of these divergent treatment strategies [7, 8, 10, 11]. Consequently, determining the optimal management approach, especially for frail patients, is often challenging. Tools for decision-making are essential to mitigate clinical complications, enhance functional outcomes, preserve quality of life, and reduce mortality.

PPF constitute a highly heterogeneous group of relatively rare fractures, making it difficult to report outcomes and study enough patients to draw robust conclusions. The PIPPAS study (Peri-Implant PeriProsthetic Survival Analysis) sought to prospectively enrol a substantial number of patients with PPF and peri-implant fractures (PIF) over a specified timeframe in Spain. The primary objective of this study was to provide insights into current trends in the management strategies for shoulder, elbow, hip, knee, or ankle PPF. Additionally, it aimed to investigate their impact on complications and mortality in the acute setting and their immediate consequences for the community. Furthermore, the study described the epidemiology, incidence, and characteristics of PPF in the Spanish population. This contemporary information on PPF management and its influence on immediate outcomes will be invaluable for addressing factors related to poorer outcomes.

## Material and methods

The PIPPAS study was a collaborative multicentre prospective observational case series study that evaluated PPF and PIF in 59 hospitals, covering 37.5% of the Spanish population (17,779,904 individuals). PPF management was the standard of care for each participating site. Patients were recruited consecutively from January 1 to December 31, 2021. Eligible patients were ≥ 18 years old and admitted with a shoulder, elbow, hip, knee, or ankle PPF. Patients with pathologic or intraoperative fractures, failed fixation without a new fracture line, or pregnancy were excluded. All patients or their relatives provided consent for their inclusion. PPF were defined as fractures occurring in a bone sustaining one component of a joint replacement. PPF were classified using the Unified Classification System (UCS) [12]. For their analysis, type D PPF were allocated to the arthroplasty group (i.e. shoulder or elbow, hip or knee, knee or ankle) that most conditioned surgical management.

The study aimed to provide valuable information about current trends in management strategies for PPF and PIF, their influence on complications and mortality in the acute setting, and their immediate impact on the community. The study also aimed to describe the epidemiology, incidence, and PPF characteristics in the Spanish population.

We enrolled 1387 patients. We collected information on patient demographics, baseline status, treatment, and hospital care based on the variables proposed in the Fragility Fracture Network (FFN) Minimum Common Dataset for hip fracture registries, which were adapted to the specific nature of PPF (Additional file 1 in Appendix S1).

The incidence of PPF was obtained from the mean annual number of elective joint replacements performed during 2019 and 2021 by all participating hospitals. Signs of X-ray loosening or a painful prosthesis prior to the PPF helped with the differentiation of B1 from B2 fractures if no intraoperative stability tests were done. Candidate predictors of in-hospital mortality, in-hospital medical complications (present or absent), weight-bearing restrictions (allowed or forbidden; only for lower limb PPF, LLPPF), and destination at hospital discharge (own home or nursing care facility) were analysed. Candidate predictors were selected according to clinical relevance. Quantitative variables were categorized based on their median values, except for the Charlson comorbidity index (CCI), clinical frailty scale (CFS), and Pfeiffer, where a significant cut-off value was used (*p* < 0.05).

The manuscript was adapted to the STROBE statement. The study coordination centre and each participating hospital obtained institutional review board approval. This study was performed following the ethical standards laid down in the 1964 Helsinki Declaration and is registered at ClinicalTrials.gov (NCT04663893).

Descriptive statistics summarized the epidemiologic data, fracture characteristics, management aspects, and in-hospital outcomes. Continuous variables were summarized as the mean and standard deviation (SD) or the median and interquartile range (IQR) as appropriate (*p* < 0.05, Shapiro–Wilk test). Categorical variables were summarized as the absolute frequencies and percentages. Relative risk was calculated with the chi-square test. Candidate predictors were analysed using a mixed-effects logistic regression model, and the results were shown as forest plots. Statistical analysis was conducted using RStudio (v.4.1.0; R Foundation for Statistical Computing, Vienna, Austria). Data were collated centrally using the REDCap data entry system (Vanderbilt University, USA) housed on secure servers at the Instituto de Estudio de Ciencias de la Salud de Castilla y León, Spain.

## Results

**Fig. 1:**
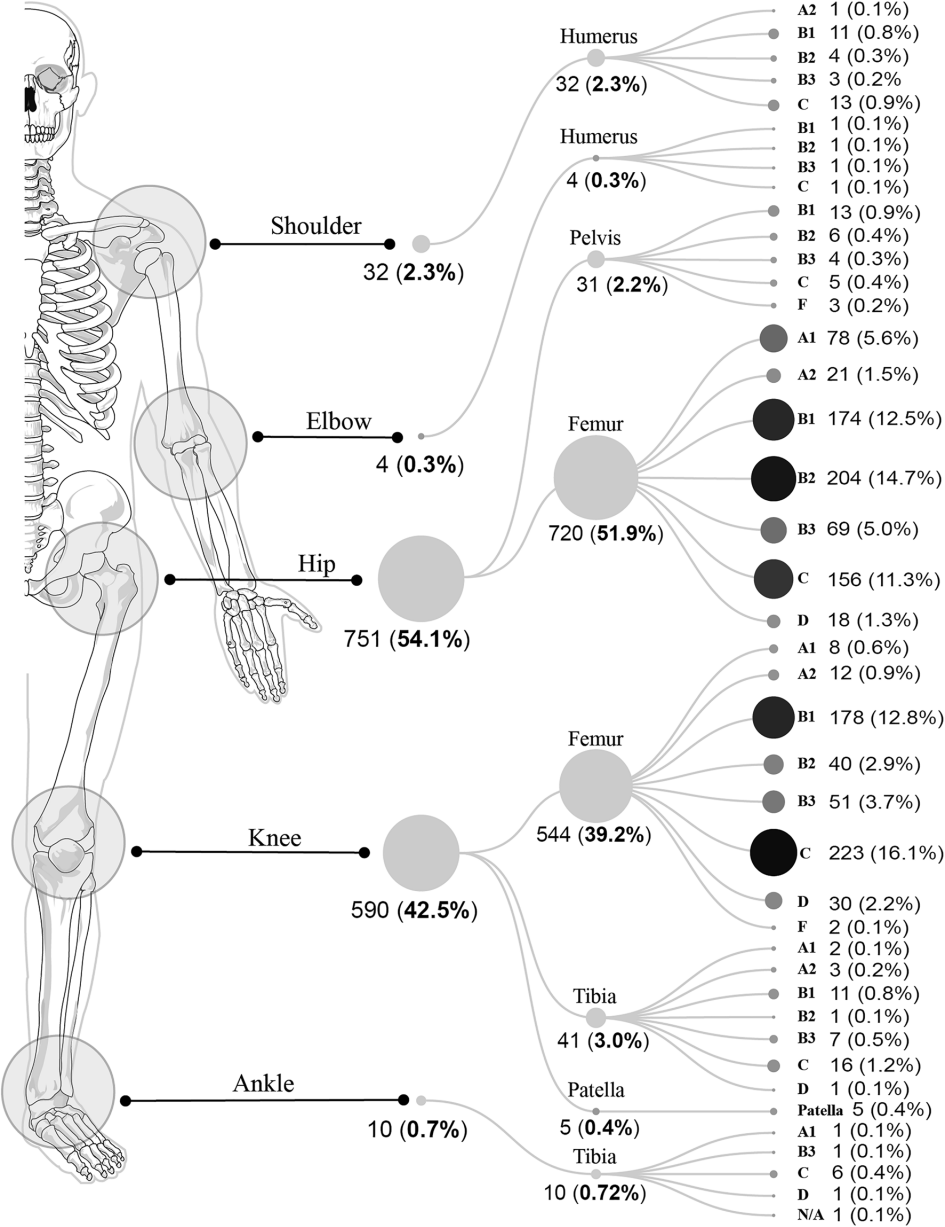
Distribution and types of PPF. The number and percentage (with respect to the total number of PPF) of fractures of each type according to the Unified Classification System (UCS) are shown. The size of each circle is proportional to the number of fractures

**Fig. 2:**
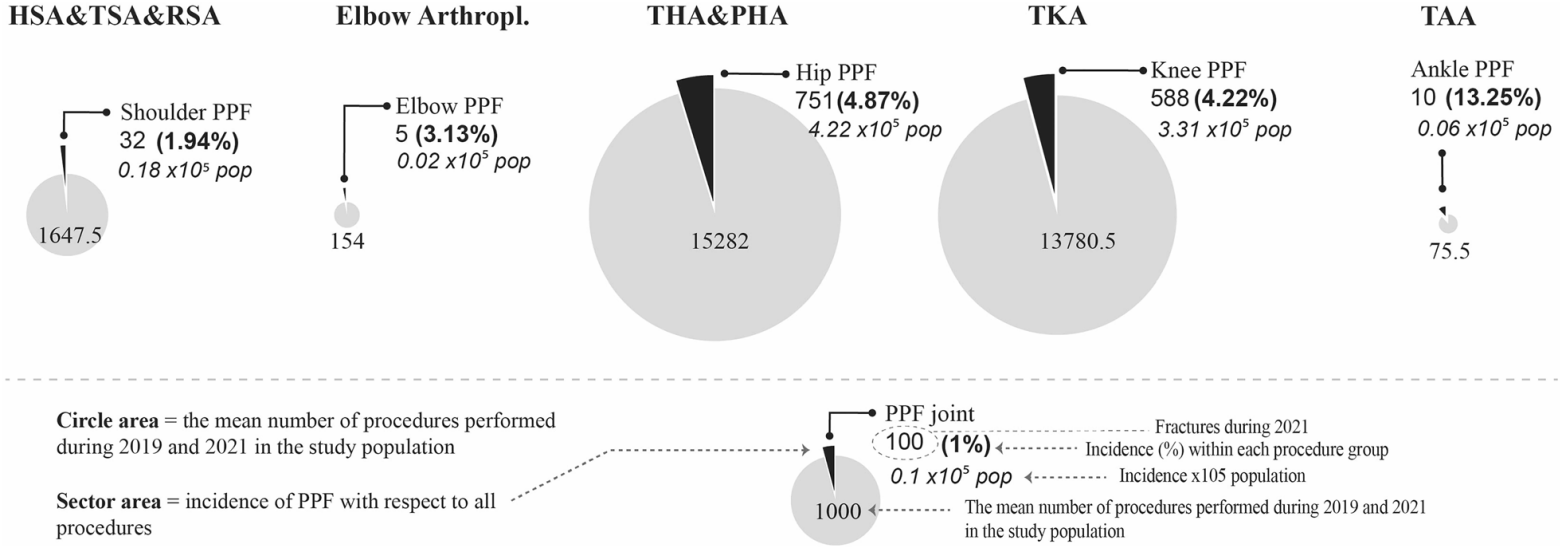
Incidence rates of shoulder PPF, elbow PPF, hip PPF, knee PPF, and ankle PPF in the Spanish population. HSA hemishoulderarthroplasty, TSA total shoulder arthroplasty, RSA reverse shoulder arthroplasty, THA total hip arthroplasty, PHA partial hip arthroplasty, TKA total knee arthroplasty, TAA total ankle arthroplasty.

The study included 32 (2.3%) shoulder, 4 (0.3%) elbow, 751 (54.1%) hip, 590 (42.5%) knee, and 10 (0.7%) ankle PPF. The PPF type distribution according to the UCS is detailed in Fig. 1. The overall incidence of PPF during 2021 was 7.80/10^5^ individuals. The estimated incidence of PPF at each location is presented in Fig. 2.

Patients were older (median 84 years, IQR 77–89), female (*n* = 1041; 75.1%), frail (median CFS 5, IQR 3–6), American Society of Anesthesiologists classification (ASA) III (52.5%), mildly cognitively impaired (median Pfeiffer 3, IQR 0–6), had at least one comorbidity (median CCI 5, IQR 4–7), were community dwellers (*n* = 1135;81.8%), and could walk outdoors (*n* = 1212; 65.6%). The patients′ demographics and baseline data are presented in Table 1. PPF diagnostic features are detailed in Table 2. Femoral components were cemented in 34% of the hip PPF and 73.6% of the knee PPF and were diagnosed as loose in 19.4% and 3.3% of the hip PPF and knee PPF patients, respectively. Femoral knee PPF most commonly occurred with uncemented femoral components (*p* < 0.01). There were no differences in the incidence of femoral hip PPF between cemented and uncemented stems (*p* = 0.06).

**Table 1:** Demographic and baseline data for patients presenting any periprosthetic fracture (PPF), humeral shoulder PPF, pelvic hip PPF, femoral hip PPF, femoral knee PPF, tibial knee PPF, and tibial ankle PPF

	PPF	Humeral shoulder	Pelvic hip	Femoral hip	Femoral knee	Tibial knee	Tibial ankle
	*N* = 1387	*N* = 32	*N* = 31	*N* = 720	*N* = 544	*N* = 41	*N* = 10
Age—years							
Median (IQR)	84 (77–89)	78 (71.75–81)	81 (72.5–85)	85 (78–90)	85 (78–90)	77 (70–84)	77 (73.25–79)
Gender—no. (%)							
Female	1041 (75.1)	26 (81.2)	21 (67.7)	477 (66.2)	466 (85.7)	36 (87.8)	7 (70)
Male	346 (24.9)	6 (18.8)	10 (32.3)	243 (33.8)	78 (14.3)	5 (12.2)	3 (30)
Place of residency—no. (%)							
Own home	1135 (81.8)	31 (96.9)	28 (90.3)	586 (81.4)	436 (80.1)	35 (85.4)	10(100)
Nursing home	229 (16.5)	0 (0)	3 (9.7)	124 (17.2)	96 (17.6)	6 (14.6)	0 (0)
Hospital	7 (0.5)	0 (0)	0 (0)	2 (0.3)	5 (0.9)	0 (0)	0 (0)
N/A	16 (1.2)	1 (3.1)	0 (0)	8 (1.1)	7 (1.3)	0 (0)	0 (0)
Pre-fracture mobility*—no. (%)							
1	385 (27.8)	25 (78.5)	13 (41.9)	178 (24.7)	147 (27)	11 (26.8)	5 (50)
2	299 (21.6)	3 (9.4)	9 (29)	164 (22.8)	113 (20.8)	6 (14.6)	2 (20)
3	225 (16.2)	1 (3.1)	2 (6.5)	141 (19.6)	71 (13.1)	8 (19.5)	2 (20)
4	287 (20.7)	0 (0)	3 (9.7)	151 (21)	120 (22.1)	12 (29.3)	0 (0)
5	177 (12.8)	2 (6.2)	4 (12.9)	78 (10.8)	89 (16.4)	4 (9.8)	0 (0)
N/A	14 (1)	1 (3.1)	0 (0)	8 (1.1)	4 (0.7)	0 (0)	1 (10)
Pfeiffer’s SPMSQ—no.							
Median (IQR)	3 (0–6)	1 (0–1.5)	2 (0–4)	3 (1–6)	3 (0–7)	0.5 (0–3)	0 (0–1)
N/A	74 (5,3)	1 (3.1)	2 (6.5)	35 (4.9)	31 (5,7)	3 (7.3)	1 (10)
CFS—no.							
Median (IQR)	5 (3–6)	3 (2.75–5)	4 (3–5.5)	5 (4–6)	5 (3–7)	5 (3–6)	3 (2.25–3)
N/A	30 (2.2)	0 (0)	0 (0)	17 (2.4)	11 (2)	1 (2.4)	0 (0)
ASA—no. (%)							
1	11 (0.8)	1 (3.1)	0 (0)	7 (1)	3 (0.6)	0 (0)	0 (0)
2	368 (26.5)	17 (53.1)	9 (29)	170 (23.6)	148 (27.2)	12 (29.3)	6 (60)
3	728 (52.5)	11 (34.4)	12 (38.7)	391 (54.3)	292 (53.7)	17 (41.5)	4 (40)
4	154 (11.1)	1 (3.1)	2 (6.5)	77 (10.7)	72 (13.2)	2 (4.9)	0 (0)
5	3 (0.2)	0 (0)	0 (0)	1 (0.1)	2 (0.4)	0 (0)	0 (0)
N/A	123 (8.9)	2 (6.2)	8 (25.8)	74 (10.3)	27 (5)	10 (24.4)	0 (0)
Charlson comorbidity index—no.							
Median (IQR)	5 (4–7)	4 (3–5)	5 (4– 6.5)	5 (4–7)	5 (4–7)	5 (3–7)	3.5 (3–4)
Osteoprotective treatment—no. (%)							
No treatment	930 (67.1)	22 (68.8)	17 (54.8)	492 (68.3)	359 (66)	29 (70.7)	6 (60)
Osteoprotective treatment^	457 (32.9)	10 (31.2)	14 (45.2)	228 (31.7)	185 (34)	12 (29.3)	4 (40)
Anti-resorptive	102 (22.3)	2 (20)	3 (21.4)	54 (23.7)	36 (19.5)	5 (41.7)	1 (25)
Bone-forming	19 (4.2)	0 (0)	1 (7.1)	9 (3.9)	8 (4.3)	1 (8.3)	0 (0)
Calcium	262 (57.3)	4 (40)	7 (50)	135 (59.2)	106 (57.3)	5 (41.7)	2 (50)
Vitamin D	379 (82.9)	8 (80)	13 (92.9)	194 (85.1)	146 (78.9)	11 (91.7)	4 (100)
Antiaggregant or anticoagulant medication—no. (%)							
None	864 (62.3)	29 (90.6)	22 (71)	431 (59.9)	339 (62.3)	29 (70.7)	9 (90)
Acenocumarol or NOAC or PAA	494 (35.6)	3 (9.4)	7 (22.6)	274 (38.1)	194 (35.7)	11 (26.8)	1 (10)
Double	19 (1.4)	0 (0)	2 (6.5)	12 (1.7)	4 (0.7)	1 (2.4)	0 (0)
N/A	10 (0.7)	0 (0)	0 (0)	3 (0.4)	7 (1.3)	0 (0)	0 (0)
Hb at admission (g/dL)—no.							
Median (IQR)	12.2 (10.9–13.4)	12.9 (11.4–13.8)	12.7 10.9–13.2)	12.2 (11–13.4)	12.1 (10.8–13.35)	12.4 (10.8–13.6)	12.3 (11.7–13.5)
N/A	19 (1.4)	0 (0)	2 (6.5)	13 (1.8)	1 (0.2)	2 (4.9)	1 (10)
Time between last prosthesis and PPF (months)—no. (%)							
< 1 month	36 (2.6)	2 (6.3)	1 (3.2)	25 (3.5)	6 (1.1)	1 (2.4)	1 (10)
From 1 to < 6 months	62 (4.5)	1 (3.1)	3 (9.7)	34 (4,7)	17 (3.1)	5 (12.2)	1 (10)
From 6 to < 12 months	33 (2.4)	0 (0)	2 (6.5)	17 (2.4)	11 (2)	2 (4.9)	1 (10)
From 1 to < 5 years	217 (15.6)	16 (50)	5 (16.1)	101 (14)	86 (15.8)	5 (12.2)	1 (10)
≥ 5 years	993 (71.6)	11 (34.4)	20 (65.4)	518 (71.9)	407 (74.8)	27 (65.9)	5 (50)
N/A	46 (3.3)	2 (6.3)	0 (0)	25 (3.5)	17 (3.1)	1 (2.4)	1 (10)

Humeral elbow and patellar PPF are not detailed (*n* = 4 and* n *= 5)

*IQR* interquartile range, *N/A* not available, *Pfeiffer′s SPMSQ* Pfeiffer′s Short Portable Mental Status Questionnaire, *CFS* clinical frailty scale, *ASA* American Society of Anesthesiologists (ASA) physical status classification system, *NOAC* new oral anti-coagulant, *PAA* platelet anti-aggregant, *Hb* haemoglobin

^*^Pre-fracture mobility scale: 1, completely independent gait; 2, independent gait outdoors with one technical aid; 3, independent gait outdoors with two technical aids; 4, independent gait indoors only, with or without aids; 5, no mobility at all or only with the help of two other people

^ Osteoprotective treatment: the percentage for each individual treatment was calculated with respect to the total number of patients receiving osteoprotective treatment

**Table 2:** Diagnostic features of periprosthetic fractures (PPF)

Joint	Shoulder 32 (2.3)	Elbow 4 (0.3)	Hip 751 (54.1)		Knee 590 (42.5)			Ankle 10 (0.7)
Bone *n* (%)	Humerus 32 (100)	Humerus 4 (100)	Pelvis 31 (4.1)	Femur 720 (95.9)	Femur 544 (92.2)	Tibia 41 (6.9)		Tibia 10 (100)
Method of fixation								
Uncemented	16 (50)	1 (25)	495 (65.9)		154 (26.1)			1 (10)
Cemented	16 (50)	3 (75)	255 (34)		434 (73.6)			9 (90)
N/A			1 (0.1)		2 (0.3)			
Presence of stem								
Stemless	0 (0)	1 (25)	5 (16.1)	64 (8.9)	449 (82.5)	24 (58.5)		2 (20)
Stem	32 (100)	3 (75)	26 (83.9)	655 (91)	94 (17.3)	17 (41.5)		8 (80)
N/A				1 (0.1)	1 (0.2)			
Infection								
Negative	29 (90.6)	4 (100)	31 (100)	694 (96.4)	526 (96.7)	37 (90.2)		8 (80)
Positive	3 (9.4)	0 (0)	0 (0)	25 (3.5)	18 (3.3)	4 (9.8)		2 (20)
N/A				1 (0.1)				
Loose prosthesis								
Negative	26 (81.2)	2 (50)	25 (80.6)	578 (80.3)	511 (93.9)	36 (87.8)		10 (100)
Positive	6 (18.8)	2 (50)	6 (19.4)	140 (19.4)	33 (6.1)	4 (9.8)		0(0)
N/A				2 (0.3)		1 (2.4)		
X-ray signs of loose prosthesis								
Negative	25 (78.1)	1 (25)	30 (96.8)	610 (84.7)	506 (93)	37 (90.2)		9 (90)
Positive	7 (21.9)	3 (75)	1 (3.2)	109 (15.1)	37 (6.8)	4 (9.8)		1 (10)
N/A				1 (0.1)	1 (0.2)			
Painful prosthesis								
Negative	22 (68.8)	3 (75)	26 (83.9)	631 (87.6)	455 (83.6)	27 (65.9)		8 (80)
Positive	10 (31.2)	1 (25)	5 (16.1)	88 (12.2)	88 (16.2)	14 (34.1)		2 (20)
N/A				1 (0.1)	1 (0.2)			
Time from arthroplasty to PPF (months)—no. (%)								
< 1 month	2 (6.3)	0 (0)	1 (3.2)	25 (3.5)	6 (1.1)	1 (2.4)		1 (10)
From 1 to < 6 months	1 (3.1)	0 (0)	3 (9.7)	34 (4.7)	17 (3.1)	5 (12.2)		1 (10)
From 6 to < 12 months	0 (0)	0 (0)	2 (6.5)	17 (2.4)	11 (2)	2 (4.9)		1 (10)
From 1 to < 5 years	16 (50)	1 (25)	5 (16.1)	101 (14)	86 (15.8)	5 (12.2)		1 (10)
≥ 5 years	11 (34.4)	3 (75)	20 (64.5)	518 (71.9)	407 (74.8)	27 (65.9)		5 (50)
N/A	2 (6.3)			25 (3.5)	17 (3.1)	1 (2.4)		1 (10)

*N/A* not available

Most patients were managed surgically (82%) under spinal anaesthesia (69%) after almost 4 days of surgical delay (92.5 IQR, 51.9–153.6 h) and through an open approach (85.9%). Femoral knee PPF were the most likely to be treated operatively (90.8%), and pelvic hip PPF were the most likely to be non-operatively managed (45.2%). The stability of prosthetic fixation was not checked in 44.1% of the patients: two-thirds of the femoral hip PPF and less than half of the femoral knee PPF cases. Prosthetic revision was performed in 33.3% of patients with femoral hip PPF, while 93.5% of femoral knee PPF received fixation. Among multiple fixation techniques, the most frequently used was a single plate (56.1%). Patients with lower limb PPF (LLPPF) managed only with fixation had a higher relative risk of being managed with restricted weight-bearing than those having their prosthesis revised (*p* < 0.01). Table 3 describes the management and surgical techniques for all PPF.

**Table 3:** Management of all periprosthetic fractures (PPF), humeral shoulder PPF, humeral elbow PPF, pelvic hip PPF, femoral hip PPF, femoral knee PPF, tibial knee PPF, and tibial ankle PPF (humeral elbow PPF and patellar PPF are not detailed;* n* = 4 and* n  *= 5, respectively)

	PPF	Humeral shoulder PPF	Pelvic hip PPF	Femoral hip PPF	Femoral knee PPF	Tibial knee PPF	Tibial ankle PPF
	*N* = 1387	*N* = 32	*N* = 31	*N* = 720	*N* = 544	*N* = 41	*N* = 10
Treatment—no. (%)							
Operative	1137 (82)	29 (90.6)	17 (54.8)	555 (77.1)	494 (90.8)	26 (63.4)	9 (90)
Non-operative	248 (17.9)	3 (9.4)	14 (45.2)	165 (22.9)	49 (9)	14 (34.1)	1 (10)
N/A	2 (0.1)				1 (0.2)	1 (2.4)	
Surgical delay (h)							
Median (IQR)	92.5 (51.9–153.6)	152.5 (82.6–208)	141.1 (65.3–210)	97.9 (56.4–157)	86.3 (48–136.4)	203.7 (96.4–288)	71.2 (45.25–120)
N/A	8 (0.7)	4 (12.5)	14 (45.2)	2 (0.3)	3 (0.6)	16 (39)	1 (10)
Type of anaesthesia							
General	251 (22.1)	26 (89.7)	8 (47.1)	139 (25)	68 (13.8)	6 (23.1)	1 (10)
Spinal	784 (69)	2 (6.9)	10 (58.8)	371 (66.8)	374 (75.7)	17 (65.4)	6 (60)
Regional	173 (15.2)	6 (20.7)	0 (0)	77 (13.9)	82 (16.6)	4 (15.4)	3 (30)
Surgical approach							
Open	745 (65.4)	27 (93.1)	15 (88.2)	437 (78.7)	238 (48.1)	16 (59.3)	6 (66.7)
MIS	233 (20.5)	2 (6.9)	1 (5.9)	69 (12.4)	155 (31.3)	3 (11.1)	2 (22.2)
PC	152 (13.3)	0 (0)	1 (5.9)	44 (7.9)	100 (20.2)	6 (22.2)	1 (11.1)
N/A	9 (0.8)	0 (0)	0 (0)	5 (0.9)	2 (0.4)	1 (3.7)	0 (0)
Direct stability check							
No	501 (44.1)	9 (31)	3 (17.6)	191 (34.4)	276 (55.9)	13 (50)	7 (77.8)
Yes, from the joint	266 (23.4)	6 (20.7)	13 (76.5)	153 (27.6)	85 (17.2)	6 (23.1)	0 (0)
Yes, from the fracture site	364 (32)	14 (48.3)	1 (5.9)	206 (37.1)	133 (26.9)	6 (23.1)	2 (22.2)
N/A	8 (0.7)	0 (0)	0 (0)	5 (0.9)	1 (0.2)	1 (3.8)	0 (0)
Cerclage for reduction							
No	611 (53.7)	13 (44.8)	12 (70.6)	156 (28.1)	397 (80.4)	23 (88.5)	8 (88.9)
Yes	520 (45.7)	16 (55.2)	5 (29.4)	394 (71)	97 (19.6)	2 (7.7)	1 (11.1)
N/A	8 (0.7)	0 (0)	0 (0)	5 (0.9)	1 (0.2)	1 (3.8)	0 (0)
Replacement							
No	887 (78)	25 (86.2)	5 (29.4)	362 (65.2)	462 (93.5)	19 (73.1)	8 (88.9)
Yes (cementless)	156 (13.7)	0 (0)	9 (52.9)	139 (25)	5 (1)	2 (7.7)	0 (0)
Yes (cemented)	88 (7.7)	4 (13.8)	3 (17.6)	49 (8.8)	27 (5.5)	4 (15.4)	1 (11.1)
N/A	8 (0.7)	0 (0)	0 (0)	5 (0.9)	1 (0.2)	1 (3.8)	0 (0)
Type of fixation							
1 plate	638 (56.1)	19 (65.5)	4 (23.5)	327 (58.9)	270 (54.7)	13 (50)	4 (44.4)
2 plates	46 (4)	4 (13.8)	1 (5.9)	6 (1.1)	27 (5.5)	4 (15.4)	3 (33.3)
Nail	194 (17.1)	1 (3.4)	0 (0)	58 (10.5)	135 (27.3)	0 (0)	0 (0)
Ex fix	4 (0.4)	0 (0)	0 (0)	0 (0)	3 (0.6)	1 (3.8)	0 (0)
Cerclage	300 (26.4)	7 (24.1)	1 (5.9)	240 (43.2)	46 (9.3)	2 (7.7)	0 (0)
Isolated screws	19 (1.7)	0 (0)	2 (11.8)	2 (0.4)	9 (1.8)	3 (11.5)	2 (22.2)
Bone graft							
No	1070(77.1)	24 (25)	14 (45.2)	515 (71.5)	479 (88.1)	25 (61)	8 (80)
Yes:	317 (22.9)	8 (75)	17 (54.8)	205 (28.5)	65 (11.9)	16 (39)	2 (20)
Strut	33 (2.9)	4 (13.8)	0 (0)	23 (4.1)	4 (0.8)	0 (0)	0 (0)
N/A	0 (0)	0 (0)	0 (0)	0 (0)	0 (0)	0 (0)	0 (0)
Overlap in mm							
Yes	653 (57.4)	20 (69)	2 (11.8)	322 (58)	290 (58.7)	12 (46.2)	4 (44.4)
Median (IQR)	87 (4 –142)	77 (53–93.5)	135 (127.5–142.5)	130 (90–161)	50 (37–75.8)	42 (20–73.25)	61.5 (59–79.25)
Gap in mm							
Yes	118 (10.4)	3 (10.3)	0 (0)	41 (7.4)	64 (13)	6 (23.1)	4 (44.4)
Median (IQR)	140 (33–200)	42 (22.5–57.5)		140 (67–200)	130.5 (23.8–189.8)	135 (75–207)	225 (186–262.5)
Kissing implants							
Yes	31 (2.7)	0 (0)	0 (0)	6 (1.1)	25 (5.1)	0 (0)	0 (0)
Surgeon experience							
> 20 replacements	316 (27.8)	7 (24.1)	10 (58.8)	206 (37.1)	84 (17)	5 (19.2)	2 (22.2)
> 20 MIPO	324 (28.5)	10 (34.5)	6 (35.3)	124 (22.3)	172 (34.8)	8 (30.8)	3 (33.3)
< 20 replacements & MIPO	533 (46.9)	14 (48.3)	3 (17.6)	247 (44.5)	248 (50.2)	12 (46.2)	5 (55.6)

Except for “treatment”, categorical variables are summarized as the absolute frequency and percentage with respect to the number of patients surgically managed in each group.

*IQR* interquartile range, *MIS* minimally invasive surgery, *PC* percutaneous, *ex fix* external fixator, *mm* millimeters, *MIPO* minimally invasive plating ostheosynthesis, *N/A* not available

The overall in-hospital mortality was 5.1%. At least one medical complication appeared in 42% of the patients: the most common complications were delirium and renal and pulmonary complications. Clinical co-management with geriatricians, internal medicine, or specialties other than anaesthesia was available for 73.9% of patients, and 78.9% required transfusion of at least one unit of packed red blood cells (cut-off level for transfusion: 7.5–8 g/dL). Regardless of the type of fracture, 77.8% of patients were initially mobilized out of bed within 24–48 h after surgery. Full weight-bearing was allowed for 34.4% of the patients with LLPPF, and 37.2% of patients went to a nursing home after discharge. Table 4 summarizes postoperative care and outcomes until hospital discharge.

**Table 4:** Postoperative care data for grouped periprosthetic fractures (PPF), humeral shoulder PPF, humeral elbow PPF, pelvic hip PPF, femoral Hip PPF, femoral knee PPF, tibial knee PPF, and tibial ankle PPF [humeral elbow (*n* = 4), patellar (*n* = 5), and tibial ankle (*n* = 10) PPF are not included]

	PPF	Humeral shoulder PPF	Pelvic hip PPF	Femoral hip PPF	Femoral knee PPF	Tibial knee PPF
	*N* = 1387	*N* = 32	*N* = 31	*N* = 720	*N* = 544	*N * = 41
In-hospital mortality						
Alive	1311 (94.5)	32 (100)	30 (96.8)	673 (93.5)	518 (95.2)	40 (97.6)
Died before surgery	24 (1.7)	0 (0)	0 (0)	19 (2.6)	5 (0.9)	0 (0)
Died in surgery	2 (0.1)	0 (0)	0 (0)	0 (0)	2 (0.4)	0 (0)
Died after surgery	46 (3.3)	0 (0)	1 (3.2)	26 (3.6)	18 (3.3)	0 (0)
N/A	4 (0.3)	0 (0)	0 (0)	2 (0.3)	1 (0.2)	1 (2.4)
Presence of medical complications during hospital stay						
No	804 (58)	27 (84.4)	22 (71)	410 (56.9)	303 (55.7)	27 (65.9)
Yes (any)	583 (42)	5 (15.6)	9 (29)	310 (43.1)	241 (44.3)	14 (34.1)
Cardiac	144 (24.7)	2 (40)	4 (44.4)	73 (23.5)	61 (25.3)	3 (21.4)
Pulmonary	163 (28)	2 (40)	4 (44.4)	93 (30)	60 (24.9)	3 (21.4)
Pulmonary thromboembolism	9 (1.5)	0 (0)	0 (0)	5 (1.6)	3 (1.2)	0 (0)
Renal	183 (31.4)	2 (40)	3 (33.3)	99 (31.9)	76 (31.5)	2 (14.3)
Cerebral	19 (3.3)	0 (0)	0 (0)	12 (3.9)	6 (2.5)	1 (7.1)
Gastrointestinal	100 (17.2)	0 (0)	0 (0)	62 (20)	36 (14.9)	2 (14.3)
Urinary tract infection	122 (20.9)	1 (20)	1 (11.1)	70 (22.6)	50 (20.7)	0 (0)
Delirium	223 (38.3)	2 (40)	2 (22.2)	116 (37.4)	99 (41.1)	4 (28.6)
In-hospital fractures	11 (1.9)	0 (0)	0 (0)	5 (1.6)	4 (1.7)	1 (7.1)
Medical staff involved in the patient care (other than trauma and anaesthesia)						
No	362 (26.1)	23 (71.9)	9 29)	178 (24.7)	120 (22.1)	19 (46.3)
Geriatrician	403 (29.1)	1 (3.1)	8 (25.8)	213 (29.6)	169 (31.1)	10 (24.4)
Internal medicine	393 (28.3)	5 (15.6)	5 (16.1)	205 (28.5)	172 (31.6)	4 (9.8)
Geriatrician and others	145 (10.5)	1 (3.1)	4 (12.9)	84 (11.7)	51 (9.4)	5 (12.2)
Others	78 (5.6)	2 (6.2)	4 (12.99	37 (5.1)	31 (5.7)	2 (4.9)
N/A	6 (0.4)	0 (0)	1 (3.2)	3 (0.4)	1 (0.2)	1 (2.4)
Initial postoperative mobilization out of bed						
< 24 h	449 (32.4)	29 (90.6)	12 (38.7)	223 (31)	154 (28.3)	17 (41.5)
24–48 h	546 (39.4)	3 (9.4)	10 (32.3)	273 (37.9)	247 (45.4)	10 (24.4)
> 48 h	353 (25.5)	0 (0)	8 (25.8)	200 (27.8)	132 (24.3)	11 (26.8)
N/A	39 (2.8)	0 (0)	1 (3.2)	24 (3.3)	11 (2)	3 (7.3)
Weight-bearing restrictions						
No restrictions	477 (34.4)	29 (90.6)	6 (19.4)	233 (32.4)	197 (36.2)	5 (12.2)
Only for transfers	177 (12.8)	1 (3.1)	5 (16.1)	105 (14.6)	64 (11.8)	2 (5.3)
Not allowed	696 (50.2)	2 (6.2)	19 (61.3)	361 (50.1)	272 (50)	31 (75.6)
N/A	37 (2.7)	0 (0)	1 (3.2)	21 (2.9)	11 (2)	3 (7.3)
Ability to walk at hospital discharge						
Yes	905 (65.2)	32 (100)	24 (77.4)	485 (67.4)	355 (65.3)	30 (73.2)
No	427 (30.8)	0 (0)	6 (19.4)	206 (28.6)	170 (31.2)	6 (14.6)
N/A	55 (4)	0 (0)	1 (3.2)	29 (4.1)	19 (3.5)	5 (12.2)
Destination at hospital discharge						
Home	787 (56.7)	29 (90.6)	18 (58.1)	407 (56.5)	293 (53.9)	24 (58.5)
Nursing home	453 (32.7)	2 (6.)	11 (35.5)	239 (33.2)	187 (34.4)	12 (29.3)
Hospital	63 (4.5)	1 (3.1)	0 (0)	26 (3.6)	33 (6.1)	3 (7.3)
N/A	84 (6)	0 (0)	2 (6.5)	48 (6.7)	31 (5.7)	2 (4.9)
Osteoprotective treatment at discharge^+^						
No treatment	669 (48.2)	21 (65.6)	10 (32.3)	348 (48.3)	253 (46.5)	26 (63.4)
Osteoprotective treatment	718 (51.8)	11 (34.3)	21 (67.7)	372 (51.7)	291 (53.5)	15 (36.6)
Anti-resorptive	264 (36.8)	1 (9.1)	6 (28.6)	144 (38.7)	105 (36.1)	7 (46.7)
Bone-forming	47 (6.5)	2 (18.2)	4 (19)	20 (5.4)	19 (6.5)	2 (13.3)
Calcium	428 (59.6)	5 (45.5)	10 (47.6)	215 (42.2)	184 (63.2)	8 (53.3)
Vitamin D	560 (78)	9 (81.8)	19 (90.5)	287 (77.2)	227 (78)	11 (73.3)
Total length of hospital stay (h)						
Median (IQR)	245 (164–370.9)	198 (129.5–295.3)	228.7 (117.1–393.7)	260 (163.5–405.4)	234.6 (166.8–336.6)	261.5 (106.5–451.3)
N/A	84 (6.1)	0 (0)	2 (6.5)	51 (7.1)	28 (5.1)	2 (4.9)
Postoperative length of hospital stay (h)						
Median (IQR)	168 (96–264)	48 (48–102)	168 (138–264)	168 (120–288)	144 (96–216)	120 (72–264)
N/A	312 (22.5)	4 (12.5)	15 (48.4)	200 (27.8)	73 (13.4)	16 (39)
Level of haemoglobin after surgical treatment						
Median (IQR)	9.6 (8.7–10.7)	10.3 (9.3–11.7)	10 (8.7–11.7)	9.7 (8.7–10.9)	9.4 (8.6–10.4)	10.3 (9.6–12–2)
N/A	91 (6.6)	5 (15.6)	5 (16.1)	51 (7.1)	15 (2.8)	9 (22)
Difference in haemoglobin level*						
Median (IQR)	2.3 (0.9–3.6)	2.4 (0.9–3.3)	1.5 (0.4–3.2)	2.2 (0.8–3.7)	2.6 (1.1–3.7)	1.7 (0.6–2.3)
N/A	91 (6.6)	5 (15.6)	5 (16.1)	51 (7.1)	15 (2.8)	9 (22)
Management of the anaemia						
No	487 (35.1)	27 (84.4)	13 (41.9)	252 (35)	158 (29)	21 (51.2)
Transfusion	710 (78.9)	3 (60)	11 (61.1)	374 (51.9)	307 (56.4)	13 (65)
Intravenous iron	389 (43.2)	2 (40)	8 (44.4)	207 (28.8)	163 (30)	8 (40)

*IQR* interquartile range; *N/A* not available

^*^Difference between haemoglobin level at admission and haemoglobin level after surgical treatment or before hospital discharge if patient was managed non-surgically. ^+^The percentages for the different osteoprotective treatments at discharge were calculated with respect to the total number of patients who were receiving osteoprotective treatment

**Fig. 3:**
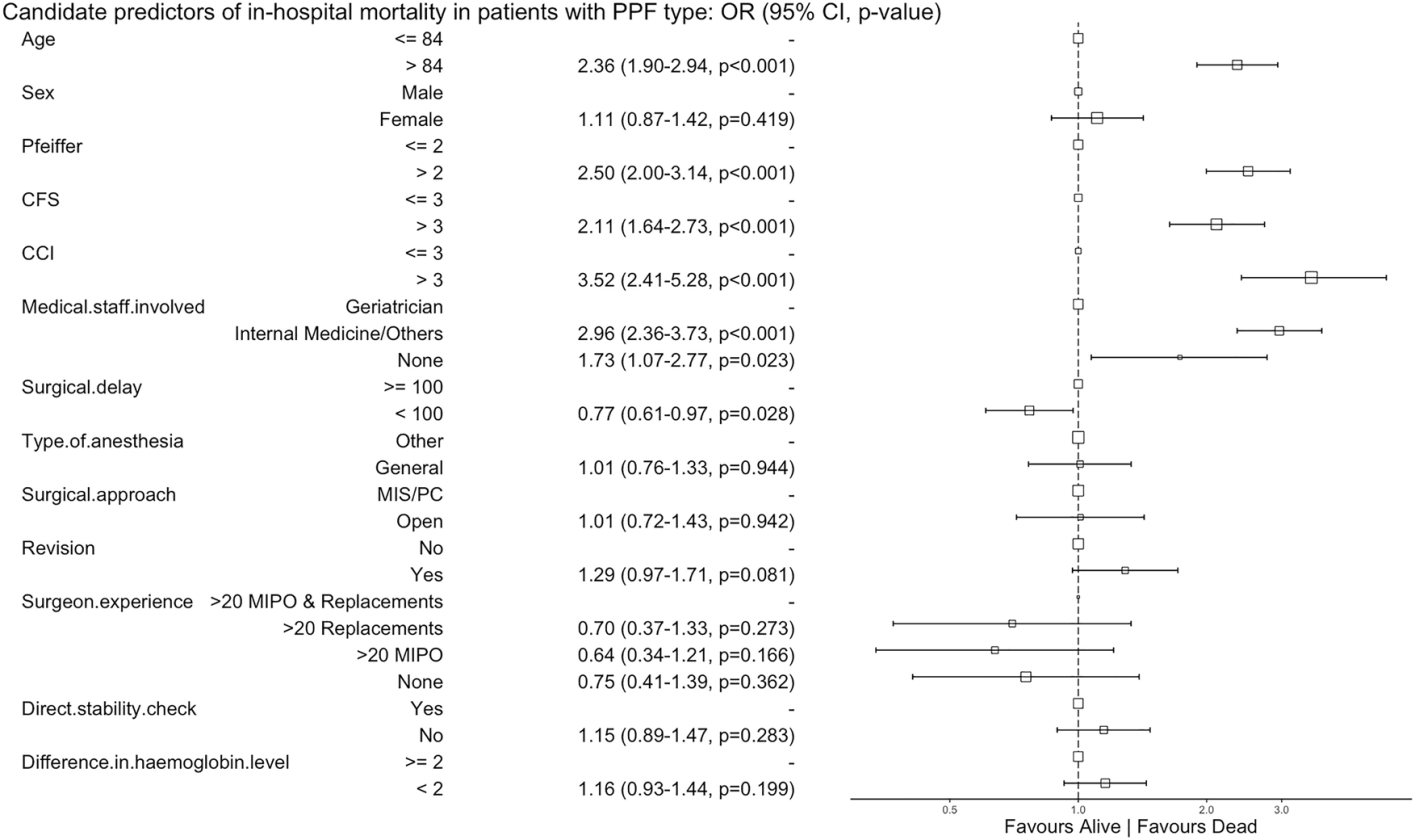
Candidate predictors of in-hospital mortality in patients with a periprosthetic fracture (PPF): alive at hospital discharge vs dies in hospital before hospital discharge. The reference category for each variable is the first value. The size of the box is proportional to the number of patients in the category.* CI* confidence interval,* OR* odds ratio,* CFS* clinical frailty scale,* CCI* Charlson comorbidity index,* MIS* minimally invasive surgery,* PC* percutaneous,* MIPO* minimally invasive plating osteosynthesis

**Fig. 4:**
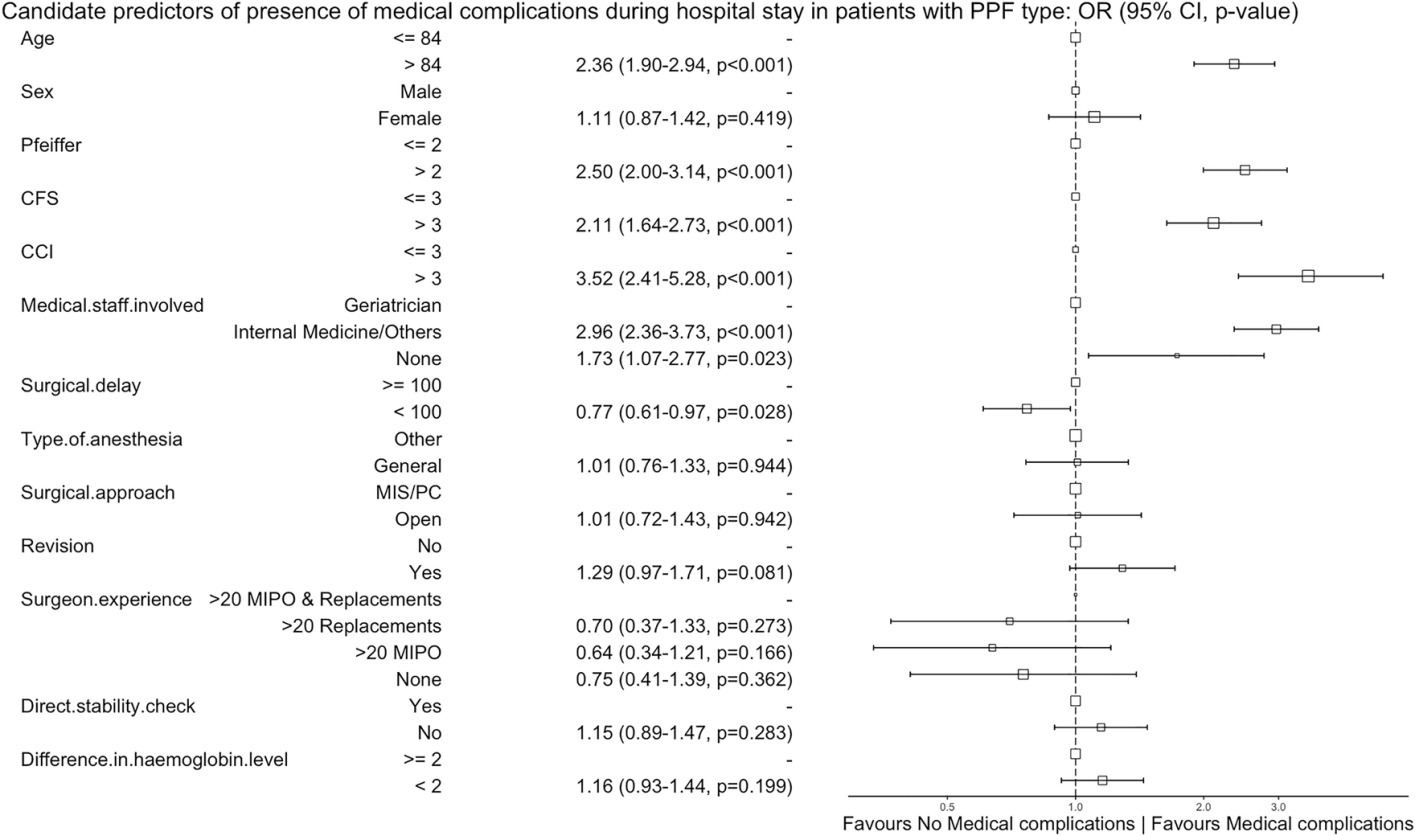
Candidate predictors of medical complications during hospital stay in patients with a periprosthetic fracture (PPF): complication vs no complications. The reference category for each variable is the first value. The size of the box is proportional to the number of patients in the category.* CI* confidence interval,* OR* odds ratio,* CFS* clinical frailty scale,* CCI* Charlson comorbidity index,* MIS* minimally invasive surgery,* PC* percutaneous,* MIPO* minimally invasive plating osteosynthesis

**Fig. 5:**
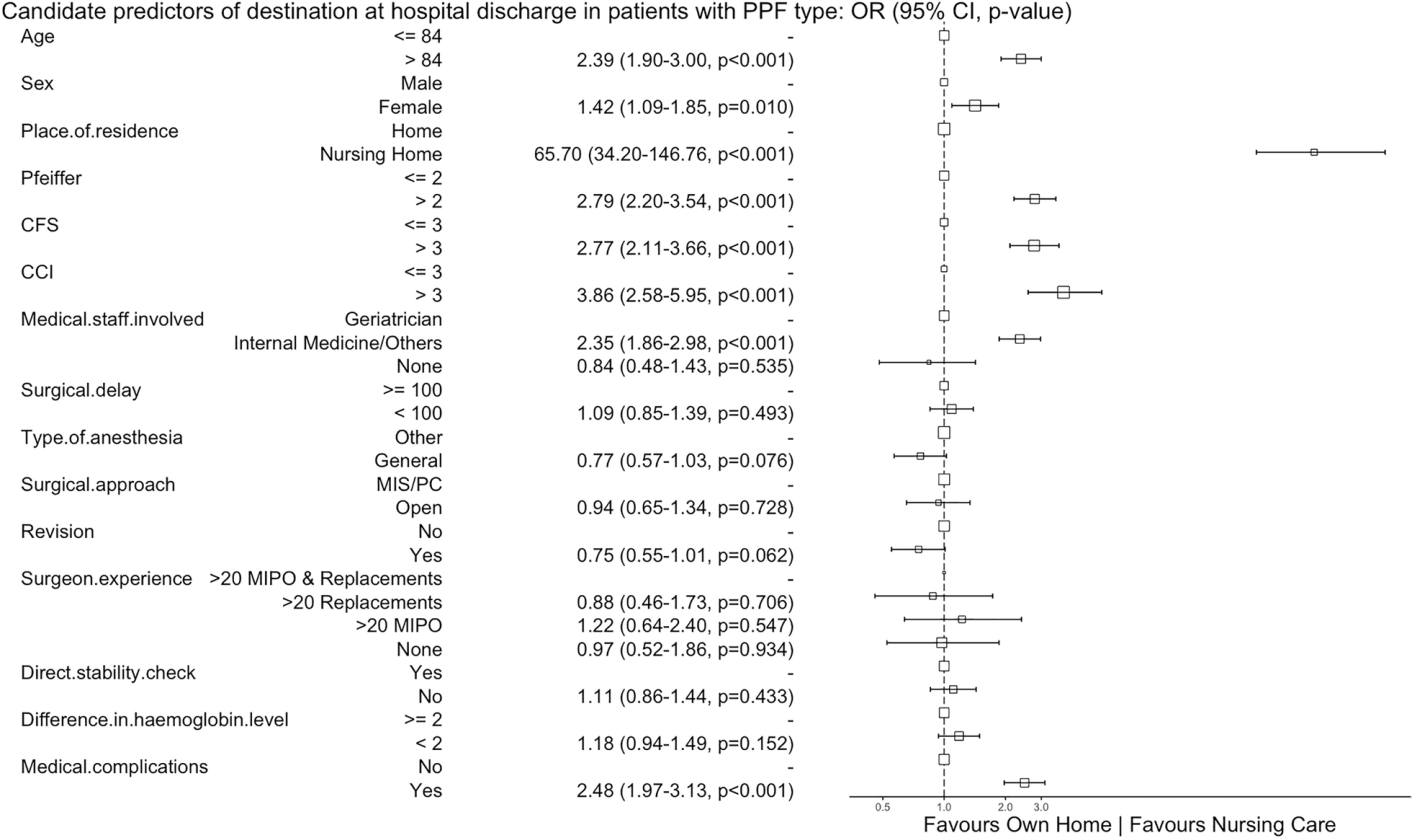
Candidate predictors of destination at hospital discharge in patients with a periprosthetic fracture (PPF): own home vs nursing care. The reference category for each variable is the first value. The size of the box is proportional to the number of patients in the category.* CI* confidence interval,* OR* odds ratio,* CFS* clinical frailty scale,* CCI* Charlson comorbidity index,* MIS* minimally invasive surgery,* PC* percutaneous,* MIPO* minimally invasive plating osteosynthesis

**Fig. 6:**
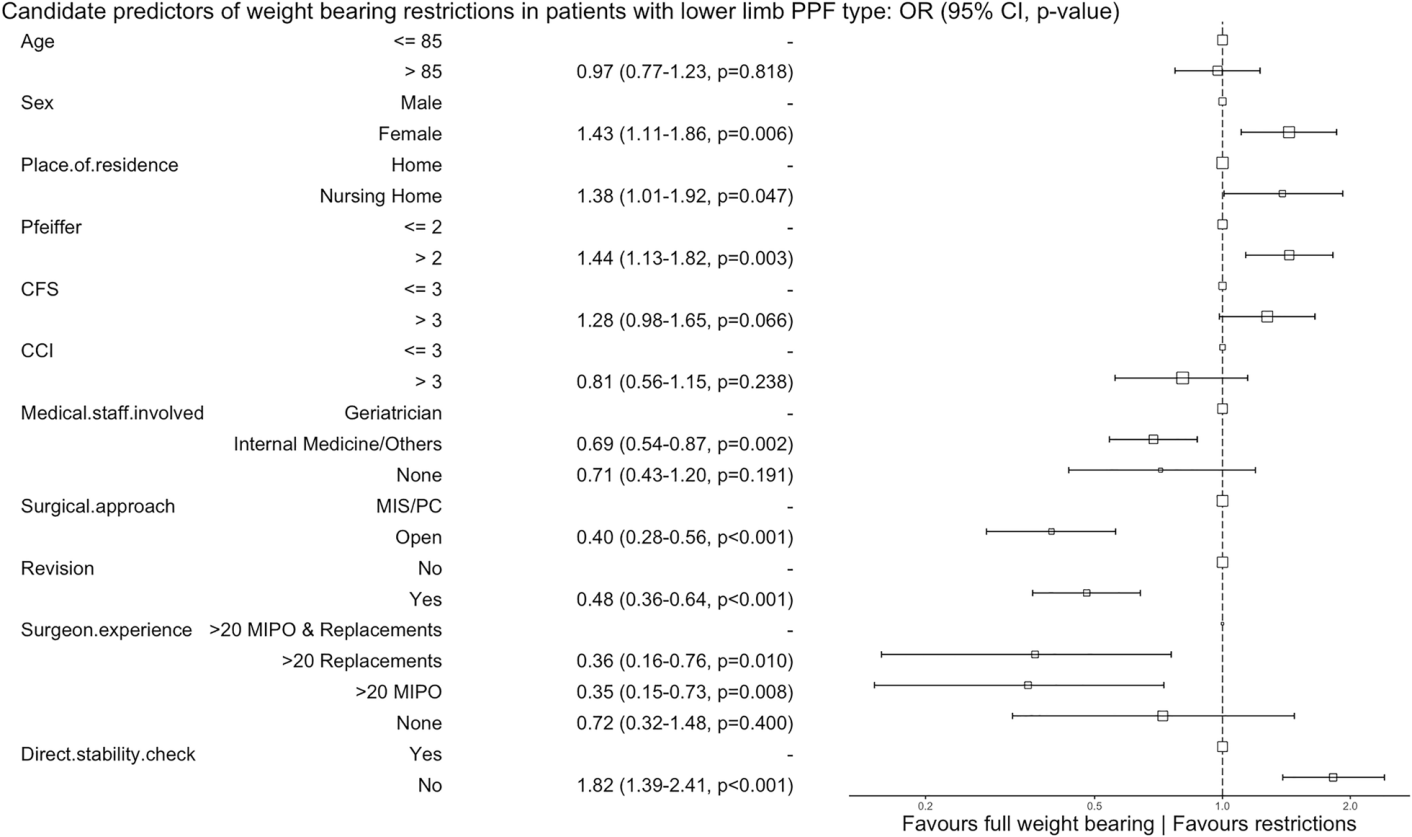
Candidate predictors of weight-bearing restrictions in patients with a lower limb [i.e. hip (pelvis and femur), knee (femur and tibia), and ankle (tibia)] periprosthetic fracture (PPF): full weight-bearing vs restrictions. The reference category for each variable is the first value. The size of the box is proportional to the number of patients in the category.* CI* confidence interval,* OR* odds ratio,* CFS* clinical frailty scale,* CCI* Charlson comorbidity index,* MIS* minimally invasive surgery,* PC* percutaneous,* MIPO* minimally invasive plating osteosynthesis

Older age (> 84 years), cognitive impairment (Pfeiffer > 2), frailty (CFS > 3), comorbidity (CCI > 3), and involvement of non-geriatric specialties favoured in-hospital mortality, medical complications, and hospital discharge to a nursing care facility (Figs. 3, 4, and 5). An operative delay of  < 100 h was associated with a reduced risk of in-hospital mortality and medical complications. Experienced surgeons, treatment with a revision prosthesis, and surgical treatment favoured full weight-bearing at hospital discharge. Weight-bearing restrictions at discharge were more common following an open approach or when the stability of the prosthesis was not checked directly (Fig. 6).

## Discussion

Despite their low incidence, PPF are severe injuries in older persons [1, 6, 10, 11]. Published data are scarce for PPF other than femoral PPF [7, 11, 13, 14]. Large sample sizes were necessary to draw conclusions regarding the current management and early outcomes of these patients [15]. To our knowledge, this is the largest specific dataset for prospectively collected PPF. The 1-year data collection period (2021) offers an up-to-date view of PPF management. The use of the minimum dataset proposed by the FNN for hip fracture registries makes future comparisons and projections possible.

The incidence and distribution of PPF and differences in sex ratios and age reflect joint replacement indications and life expectancy, which vary among cultures and populations [16]. Most series reported a mean age of 64–78 years [2, 14, 17], which was younger than our population. The femur was the most common location (91%) because THA and TKA are the most implanted joint replacements overall [18, 19].

Data related to patients′ pre-fracture health status are usually limited to the ASA scores. Information on CCI, mobility, references to osteoporosis treatment, or place of residence documentation are scarce in previous studies. PIPPAS demonstrated that CFS, CCI, and cognitive impairment were related to poorer immediate outcomes. In frail patients, CFS, CCI, and cognitive status can help complications to be addressed and an adequate surgical strategy to improve functional weight-bearing and to minimize the social burden at hospital discharge to be selected. The percentage of patients with an ASA II score ranges across studies from 24% (in our study) to 54% [11, 20], which is probably influenced by differences in age.

The distribution of femoral hip PPF is similar in other published series: around 15% were type A, 70% type B, and 15% type C [2, 21]. The exception is the Mayo Clinic′s series, where 34.7% of the patients were type A1 [22]. In 24 patients, Liu et al. found a higher percentage of type C tibia knee PPF than we did: 70.8% vs 39% [14]. According to the Mayo Clinic′s series and the review by Carli, postoperative femoral hip PPF were most common with uncemented stems [15, 22]; the same is true for hemiarthroplasty [23], which showed different incidences depending on the stem design [22]*.* In our study, as in that of Karam et al., femoral hip PPF occurred equally in cemented and uncemented stems (*p* = 0.06) [24]. We found that femoral knee PPF were more common with uncemented femoral components than with cemented ones (*p* < 0.01), although Nugent et al. found no difference [25]. Detailed analysis of the implant design and fixation method in each joint and type of PPF are needed to clarify their contributions to the PFF risk.

In-hospital mortality for femoral hip PPF ranges from 2.4% to 8% [22, 27]. This variation may be due to the patients’ age and comorbidities, as age and frailty variables favoured in-hospital mortality in our study. Patients managed by a geriatrician had a lower risk of medical complications and in-hospital mortality and a greater chance of returning to the community at hospital discharge (*p* < 0.05). Therefore, as suggested [1], multidisciplinary co-management starting at diagnosis should be organized to benefit patients with PPF and address potential complications promptly.

A wide range of surgical strategies can be applied to each PPF type, and this range of strategies depends on the PPF type considered [1, 2, 6, 7, 9, 11, 13, 14, 20]. Surgical management can be grouped into revision arthroplasty or internal fixation. Revision to a long stem is recommended for all type B2 femoral hip PPF, especially transverse patterns [3, 28, 29]. Other series had higher revision rates than seen in the current study: 60.9–86.8% for femoral hip PPF and 19.3% for femoral knee PPF [1, 11, 14]. Recent publications show a trend towards considering internal fixation in Vancouver B2 and B3 fractures [1, 2, 6, 7, 9, 13, 20]. PIPPAS showed that revision arthroplasty favoured full weight-bearing and hospital discharge to the patient’s own home (*p* < 0.05), but revision hip arthroplasty for PPF is associated with a high risk of mortality (*p* < 0.05) [13], as revealed in this study. We found that the recommendation for routine intraoperative stem stability tests before fixation was not followed, as the stability of one-third of femoral stems was not checked. However, not checking the stability of the prosthesis favoured weight-bearing restrictions (*p* < 0.05). The reason for this might be that when the stability is checked, the most common surgical strategy is prosthesis revision, which favours full weight-bearing. When the surgical strategy is fixation, usually with plates, surgeons might not feel confident with full weight-bearing. Compared to revision of the prosthesis, patients managed only with fixation showed a higher relative risk of restricted weight-bearing (*p* < 0.01). There is a tendency to use double plating techniques to increase stability to allow full weight-bearing, although they are not widely used. Anatomical polyaxial locking plates allow less invasive surgical approaches and the placement of locking screws around the stem, thus providing a certain degree of stability to a loose prosthesis [4]. Further analysis of the influence of full weight-bearing on failure of fixation rates in LLPPF managed only with fixation should follow. Therefore, our suggestion is to individualize every case, taking into consideration how frail the patient is and the surgical strategy options that could be used to achieve the best functional outcome in each scenario.

Open approaches were related to restricted weight-bearing for at least 30 days postoperatively (*p* < 0.05), but many authors mainly used open approaches for fixation [3, 6, 7, 13, 17], even though hypoaggressive approaches are recommended for PPF fixation [4, 26]. Surgeon experience favoured full weight-bearing (*p* < 0.05). Competency in the management of PPF could help improve in-hospital outcomes.

The operative delay varies from 6.06 to 4.07 days in published series [6, 11, 21, 31, 32]. Johnson-Lynn found no association between surgical delay and inpatient mortality [5], but we observed that surgery within the first 100 h (4.17 days) favoured survival and reduced the risk of medical complications (*p* < 0.05). Patients who are fit for surgery may benefit from prompt surgical management, and co-management may improve their medical condition, limiting the influence of comorbidities on survival and complications.

There is limited information regarding the type of anaesthesia in the management of PPF. Haughom et al. reported that 83.4% of patients with femoral hip PPF underwent surgery under general anaesthesia [32], in contrast to 13.8% and 25% of patients with femoral knee and femoral hip PPF in the PIPPAS study. However, the type of anaesthesia did not influence in-hospital outcomes in PPF.

Strut grafts have been widely used in the management of PPF [22], although there is a trend towards limiting their use: 1.4% in COMPOSE [11] and 2.2% in PIPPAS. This may be attributed to the increased use of hypoaggressive approaches and double-plate fixation when additional stability is required.

Non-operative management for femoral PPF is limited to selected cases, with reported rates ranging from 0 to 33% [11, 30]. Non-operative treatment was a candidate predictor of in-hospital mortality and worse outcomes (*p* < 0.05). It remains unclear whether the indication for non-surgical treatment was driven by the patient′s comorbidities or the fracture pattern. Nevertheless, it is generally accepted that patients with femoral fractures benefit from surgery [4, 33].

Early weight-bearing is a crucial factor in limiting the impact of LLPPF on functional outcomes and the return to the community [34, 35]. Restricted weight-bearing is associated with limited possibility of returning to the community, resulting in economic and social burdens [34, 35]. Frailty variables correlated with weight-bearing restrictions and cannot be modified. However, the operative technique can be improved to allow unrestricted weight-bearing, facilitating early functional recovery and social independence [3].

This study has several limitations, including the following. (1) The heterogeneity of PPF—although multiple surgical strategies were employed, the population, management, and outcomes were quite similar. (2) Specific details of and differences between fracture types and their surgical treatments were not explored, so further analysis should follow. A comprehensive understanding of these fractures can assist readers in organizing their resources. (3) Participating sites were responsible for data accuracy. (4) The stability of prosthesis fixation was always determined by the treating site. (5) Candidate predictors provide useful information about the potential correlation between two variables, but they cannot be used to determine casuality.

## Conclusions

PPF patients are frail: CFS > 3, CCI > 3 and mild cognitive impairment (Pfeiffer > 2) are associated with higher morbidity and mortality rates in the acute setting. Hospital discharge to a nursing home and weight-bearing restrictions are common outcomes in such cases. Surgical strategies directly influence these immediate outcomes. However, current arthroplasty fixation check and revision rates do not adhere to established guidelines. Nevertheless, revision arthroplasty and experienced surgeons were associated with fewer weight-bearing restrictions. A surgical delay exceeding 100 h and a lack of co-management with geriatricians were linked to in-hospital mortality and medical complications. Hypoaggressive approaches favoured full weight-bearing and are therefore recommended for fixation. The PIPPAS study provides insights into potential risk factors, which can aid in the development of individualized management strategies for the benefit of patients with PPF.

### Supplementary information

**Additional file 1.** Data collected from patients presenting with a Periprosthetic fracture.

## Data Availability

The datasets used and/or analysed during the current study are available from the corresponding author on reasonable request.

## Abbreviations

PPF: Periprosthetic fractures
UCS: Unified Classification System
PIPPAS: Peri-Implant PeriProsthetic Survival Analysis
THA: Primary total hip arthroplasty
TKA: Total knee arthroplasty
IQR: Interquartile range
SD: Standard deviation
ASA: American Society of Anesthesiologists physical status classification system
CCI: Charlson comorbidity index
CFS: Clinical frailty scale
LL: Lower limb
