# Supplementary material for: Optimizing periprosthetic fracture management and in-hospital outcome: insights from the PIPPAS multicentric study of 1387 cases in Spain
Source: J Orthop Traumatol. 2024 Mar 7;25:13. doi: 10.1186/s10195-024-00746-6 (PMC10920552; doi:10.1186/s10195-024-00746-6)
Supplement: Supplementary file 1 — Additional file 1. Data collected from patients presenting with a Periprosthetic fracture. [file 10195_2024_746_MOESM1_ESM.docx]

**SUPLEMENTARY MATERIAL**

**Data collected from patients presenting with a Periprosthetic fracture**

**BLOCK 1. Epidemiology, Health status, and in-hospital management variables:**

1.1 Date of birth

1.2 Gender

1.3 Pre-fracture place of residence: own home, nursing facility, acute hospital, N/A

1.4 Pre-fracture mobility: (FFN-MCD scale)

1 completely independent gait

2 outdoors independent gait with 1 technical aid

3 outdoors independent gait with 2 technical aids

4 only indoors independent gait w or w/o aids

5 no mobility at all or with the help of 2 other people

6 N/A

1.5 Mental assessment: Pfeiffer´s SPMSQ Pfeiffer´s Short Portable Mental Status Questionnaire. Number of mistakes

1.6 Clinical Frailty Scale (CFS) (2 weeks pre-fracture).

1.7 ASA: I, II, III, IV, V, N/A

1.8 Charlson comorbidity index (CCI): Individualized organ/system punctuations are registered

1.9 Type of fracture: Periprosthetic, Peri-implant

If the bone hosts one prosthesis and one fixation device, choose the type of fracture that most influences on the treatment.

1.10 Are there more implants in the same bone? No, Prosthesis, Nail, Plate, Isolated screw.

1.11 Total number of fractures supported in the injured bone (including the actual fracture)?

1.12 Osteoprotective treatment: Anti-resorptive, Bone-forming, Calcium, Vitamin D, none.

1.13 Antiaggregant or anticoagulant medication:

1 NO,

2 Acenocumarol, NOAC, PAA (Clopidogrel / Ticlo / AAS 300) NOAC: New Oral Anti-Coagulant, PAA: Platelet Anti-Aggregant

3 Double

1.14 Date of Fracture

1.15 Date and time the patient is admitted in emergency

1.16 Does the patient receive surgical treatment? Yes or No

Date and time of surgical treatment

1.17 Haemoglobin level (g/dL): At admission and 1^st^ day post-op

1.18 Medical Complications during hospital stay: (which may need treatment)

Cardiac, respiratory, Pulmonary thromboembolism, Urinary infection, Renal, Brain, delirium, Gastro-intestinal, in-hospital fractures, None

(Multiple answers are possible except none)

1.19 Co-management with other specialties: (apart from traumatology and anaesthesia): Geriatrics, Internal Medicine, other specialties, Geriatrics and Other specialties, None

1.20 ¿Did the patient sit down during the first day post-op? If the patient was managed non-surgically, did the patient sit the day after the decision? Yes or No

1.21 Was full weight bearing allowed?

No restrictions or with external aids in elderly patients

Only for transferences

Complete restriction (wheelchair in elderly patients or two crutches in young patients)

1.22 Was the patient walking at hospital discharge? Yes or No (either with or without weight bearing restrictions)

1.23 In-hospital Mortality: Alive, dies before surgical treatment, dies in the operation room, dies post-operatively

1.24 Hospital discharge: Date and Time

1.25 Destination at hospital discharge:

Own home

Healthcare institution

Acute hospital

N/A

1.26 Osteoprotective treatment at hospital discharge: Anti-resorptive, Bone-forming, Calcium, Vitamin D, none. (Multiple answers are possible except none)

**BLOCK 2. DIAGNOSIS Variables**

2A Periprosthetic fractures diagnosis

2A.1 USP Classification:

2A.1.1 Bone:

Humerus Scapula Forearm Pelvis Femur Tibia Patella

2A.1.2 Joint:

Shoulder Elbow Hip Knee Ankle

2A.1.3 Type:

A1 A2 B1 B2 B3 C D F

2A.2 Date when the prosthesis was implanted

2A.3 Previous Infection? Yes or No

2A.4 Was the prosthesis loose previously? Yes or No

2A.5 Is the prosthesis cemented? Yes or No

2A.6 Was the prosthesis painful previously? Yes or No

2A.7 Were there radiological signs of loosening previously? Yes or No

2A.8 Does the prosthesis have a stem? Yes or No

2A.9 Does the bone host a hip arthroplasty? No Yes, stem prosthesis Yes, stemless prosthesis

2A.10 Does the bone host a knee arthroplasty? No Yes, stem prosthesis Yes, stemless prosthesis

**BLOCK 3. TREATMENT Variables**

3A PeriProsthetic Fracture Treatment

3A.1 Approach: Percutaneous MIS-Hypo-invasive Open

(Percutaneous: as for a small incision for a nail; Hypo-invasive: several incisions of the minimum size needed)

3A.2 Was the stability/fixation of the prosthesis checked?

Yes, from the joint Yes, from the fracture site No

3A.3 Was a cerclage used as a reduction tool? Yes or No

3A.4 Was revision of the prosthesis the treatment option? No Yes, cementless Yes, cemented

3A.5 Was fixation the treatment option? No Yes, 1 Plate Yes, 2 Plates Yes, Nail

Yes, definitive external fixator Yes, cerclage Yes, isolated screws (Multiple answers option)

3A.6 Is there overlapping between implants? And length in milimetres

Overlap + Kissing 0 Gap – ___ mm

3A.7 Was bone graft used? No Yes, Strut Yes, cancellous/reaming product

3A.8 Surgeon experience? >20 arthroplasty revisions in the last 12 months >20 MIPO surgeries in the last 12 months None of the previous (multiple answers are possible except none)

3A.9 Anaesthesia? General Neuro-axial Regional Different form previous

**BLOCK 4. 30 DAYS FOLLOW-UP** (from surgical treatment or from diagnosis if non-surgical treatment)

4A.1 Alive at 30 days follow-up? Yes or no

4A.1.2 Date of death

4A.2 Is weight bearing allowed?

No restrictions or with external aids in the elderly

Only for transferences

Weight bearing is forbidden (wheelchair in the elderly or crutches in young patients)

4A.3 Mobility at 30 days follow up: (FFN-MCD scale)

1 completely independent gait

2 outdoors independent gait with 1 technical aid

3 outdoors independent gait with 2 technical aids

4 only indoors independent gait w or w/o aids

5 no mobility at all or with the help of 2 other people

6 N/A

4A.4 Any medical complication needing hospital admission within 30 days post-op?

☐NO ☐Heart ☐Respiratory ☐Pulmonary thromboembolism ☐Renal ☐Cerebral ☐Gastro-intestinal ☐Any other

(Multiple answers are possible except none)

4A.5 Surgical complications at 30 days follow up:

☐NO ☐Fracture in the same bone ☐Fixation failure ☐Dislocation

☐Loosen prosthesis ☐Infection (Multiple answers are possible except none)

4A.6 Place of residence at 30 days follow up: own home, nursing facility, acute hospital, N/A

4A.7 Osteoprotective treatment at 30 days follow up: Anti-resorptive, Bone-forming, Calcium, Vitamin D, none. (Multiple answers are possible except none)

**BLOCK 5. 6 MONTHS FOLLOW-UP** (from surgical treatment or from diagnosis if non-surgical treatment)

5A.1 Alive at 6 months follow-up? Yes or no

5A.1.2 Date of death

5A.2 Quality of Life questionnaire EQ5D:

5A.2.1 Mobility:

5A.2.2 Personal care:

5A.2.3 Household chores:

5A.2.4 Pain/ discomfort:

5A.2.5 Anxiety/Depression:

5A.3 Mobility at 6 months follow up: (FFN-MCD scale)

1 completely independent gait

2 outdoors independent gait with 1 technical aid

3 outdoors independent gait with 2 technical aids

4 only indoors independent gait w or w/o aids

5 no mobility at all or with the help of 2 other people

6 N/A

5A.4 Clinical Frailty Scale (CFS)

5A. Any medical complication needing hospital admission within 6 months post-op?

☐NO ☐Heart ☐Respiratory ☐Pulmonary thromboembolism ☐Renal ☐Cerebral ☐Gastro-intestinal ☐Any other

(Multiple answers are possible except none)

5A.6 Surgical complications at 30 days follow up:

☐NO ☐Fracture in the same bone ☐Fixation failure ☐Dislocation

☐Loosen prosthesis ☐Infection (Multiple answers are possible except none)

5A.7 Is the fracture healed? Yes No Non-applicable (treated with a prosthesis)

5A.8 Place of residence at 6 months follow up: own home, nursing facility, acute hospital, N/A

5A.9 Osteoprotective treatment at 30 days follow up: Anti-resorptive, Bone-forming, Calcium, Vitamin D, none. (Multiple answers are possible except none)

**BLOCK 6. 12 MONTHS FOLLOW-UP** (from surgical treatment or from diagnosis if non-surgical treatment)

6A.1 Alive at 12 months follow-up? Yes or no

6A.1.2 Date of death

6A.2 Quality of Life questionnaire EQ5D:

6A.2.1 Mobility:

6A.2.2 Personal care:

6A.2.3 Household chores:

6A.2.4 Pain/ discomfort:

6A.2.5 Anxiety/Depression

6A.3 Mental assessment: Pfeiffer´s SPMSQ Pfeiffer´s Short Portable Mental Status Questionnaire. Number of mistakes

6A.4 Mobility at 12 months follow up: (FFN-MCD scale)

1 completely independent gait

2 outdoors independent gait with 1 technical aid

3 outdoors independent gait with 2 technical aids

4 only indoors independent gait w or w/o aids

5 no mobility at all or with the help of 2 other people

6 N/A

6A.5 Clinical Frailty Scale (CFS)

6A.6 Any medical complication needing hospital admission within 6 months post-op?

☐NO ☐Heart ☐Respiratory ☐Pulmonary thromboembolism ☐Renal ☐Cerebral ☐Gastro-intestinal ☐Any other

(Multiple answers are possible except none)

6A.7 Surgical complications at 30 days follow up:

☐NO ☐Fracture in the same bone ☐Fixation failure ☐Dislocation

☐Loosen prosthesis ☐Infection (Multiple answers are possible except none)

6A.8 Is the fracture healed? Yes No Non-applicable (treated with a prosthesis)

6A.9 Place of residence at 12 months follow up: own home, nursing facility, acute hospital, N/A

6A.10 Osteoprotective treatment at 12 months follow up: Anti-resorptive, Bone-forming, Calcium, Vitamin D, none. (Multiple answers are possible except none)

N/A: Non-Available, CFS: clinical frailty scale, ASA: American Society of Anesthesiologists (ASA) physical status classification system, NOAC: New Oral Anti-Coagulant, PAA: Platelet Anti-Aggregant, Hb: Haemoglobin.
